# Recent advances in the formulation of PLGA microparticles for controlled drug delivery

**DOI:** 10.1007/s40204-020-00139-y

**Published:** 2020-10-15

**Authors:** Elena Lagreca, Valentina Onesto, Concetta Di Natale, Sara La Manna, Paolo Antonio Netti, Raffaele Vecchione

**Affiliations:** 1 Center for Advanced Biomaterials for HealthCare@CRIB, Istituto Italiano di Tecnologia, Largo Barsanti e Matteucci 53, 80125 Naples, Italy; 2 Department of Pharmacy, CIRPEB: Centro Interuniversitario di Ricerca sui Peptidi Bioattivi, University of Naples “Federico II”, Via Mezzocannone 16, 80134 Naples, Italy; 3 Interdisciplinary Research Center of Biomaterials, CRIB, University Federico II, P.leTecchio 80, 80125 Naples, Italy; 4 Department of Chemical, Materials and Industrial Production Engineering (DICMaPI), University of Naples Federico II, P.le Tecchio 80, 80125 Naples, Italy

**Keywords:** PLGA MPs, Double emulsion, Single emulsion, Drug encapsulation, Drug release

## Abstract

Polymeric microparticles (MPs) are recognized as very popular carriers to increase the bioavailability and bio-distribution of both lipophilic and hydrophilic drugs. Among different kinds of polymers, poly-(lactic-*co*-glycolic acid) (PLGA) is one of the most accepted materials for this purpose, because of its biodegradability (due to the presence of ester linkages that are degraded by hydrolysis in aqueous environments) and safety (PLGA is a Food and Drug Administration (FDA)-approved compound). Moreover, its biodegradability depends on the number of glycolide units present in the structure, indeed, lower glycol content results in an increased degradation time and conversely a higher monomer unit number results in a decreased time. Due to this feature, it is possible to design and fabricate MPs with a programmable and time-controlled drug release. Many approaches and procedures can be used to prepare MPs. The chosen fabrication methodology influences size, stability, entrapment efficiency, and MPs release kinetics. For example, lipophilic drugs as chemotherapeutic agents (doxorubicin), anti-inflammatory non-steroidal (indomethacin), and nutraceuticals (curcumin) were successfully encapsulated in MPs prepared by single emulsion technique, while water-soluble compounds, such as aptamer, peptides and proteins, involved the use of double emulsion systems to provide a hydrophilic compartment and prevent molecular degradation. The purpose of this review is to provide an overview about the preparation and characterization of drug-loaded PLGA MPs obtained by single, double emulsion and microfluidic techniques, and their current applications in the pharmaceutical industry.

**Graphic abstract**
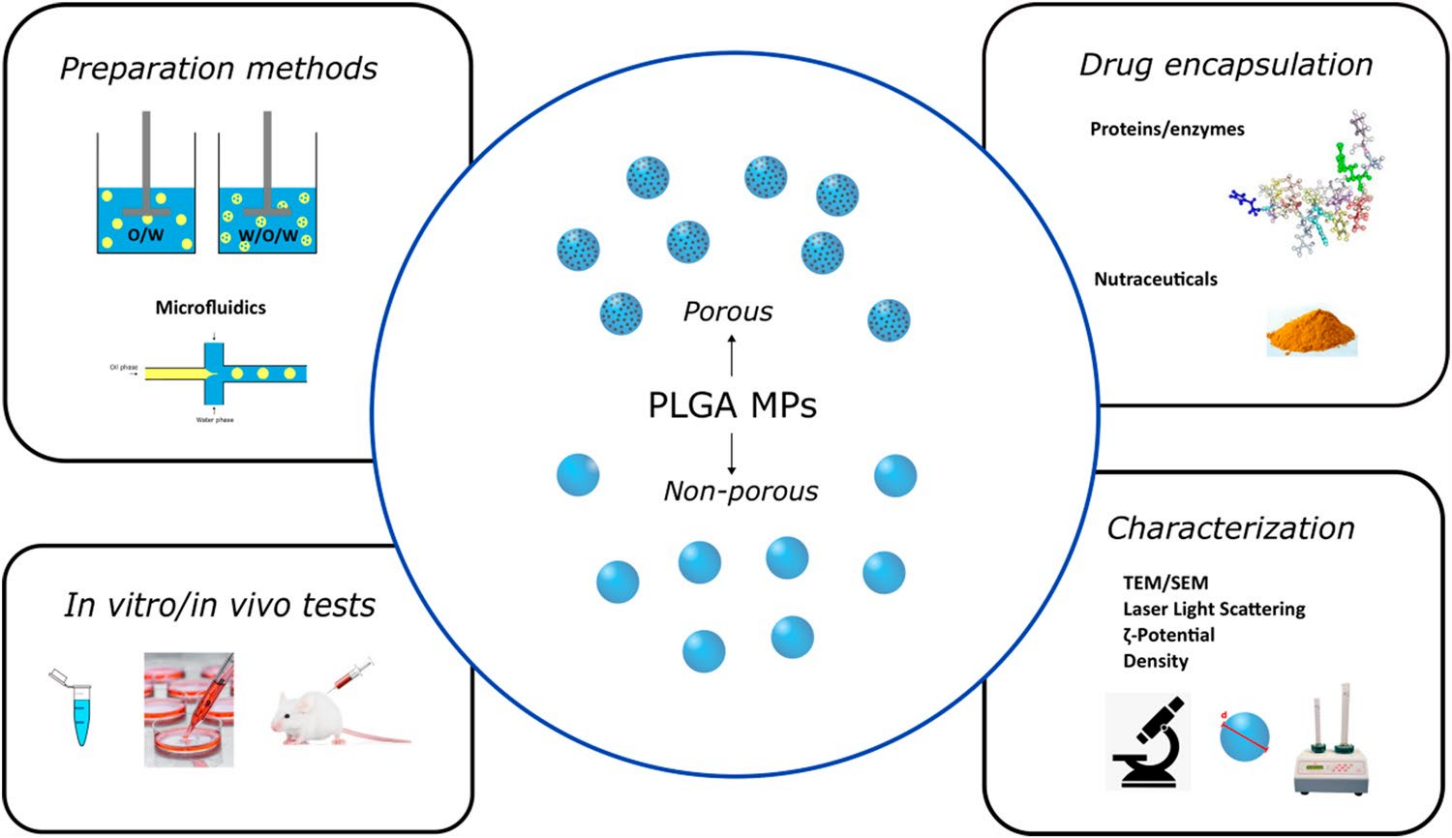

## Introduction

**Fig. 1:**
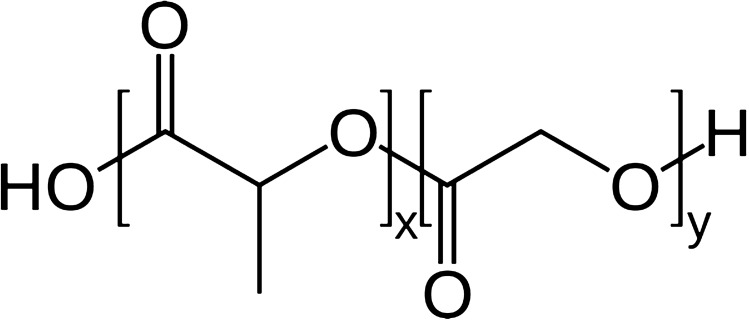
PLGA structure (*x* number of monomer of lactic acid, *y* number of monomer of glycolic acid)

Polymeric microparticles (MPs) are gaining more and more interest not only as drug delivery systems (Karp et al. 2019), but also in biosensing and tissue engineering (Martins et al. 2018; Qodratnama et al. 2015). This success is mostly due to the many advantages of this kind of particles including relatively simple procedures and possible industrial scale-up (Chung et al. 2019). As a drug delivery system, MPs offer many advantages such as the use of different administration routes or the opportunity of encapsulating different molecules including proteins (Ospina-Villa et al. 2019) and nucleic acid (McKiernan et al. 2018). In particular, MPs can be used for the controlled release of drugs (Choi et al. 2011; Guo et al. 2015) that can be modulated by choosing the kind of polymer and its chemical and molecular features such as molecular weight (MW), monomer composition (Takeuchi et al. 2017), crystallinity, glass transition temperature (*T*_g_), and inherent viscosity (Ansary et al. 2014). In this scenario, a lot of biodegradable polymers can be used to formulate MPs as alginate (Strobel et al. 2020), dextran (Shah et al. 2019), chitosan (Batista et al. 2019), gelatin (da Silva and Pinto et al. 2019) and poly-(lactic-*co*-glycolic acid) (PLGA) (Kapoor et al. 2015). These polymers are preferred, because their degradation products are non-toxic metabolites and are also easily eliminated (Makadia and Siegel 2011). Here, the focus is on a random copolymer of poly-(glycolic acid) (PGA) and poly-(lactic acid) (PLA) (Fig. 1), which employment is mostly due to the possibility to achieve a controlled drug release by governing its bio-degradation which is ruled by the polymer chemistry such as glycoside units content, initial MW (Amoyav et al. 2019; Xia, Li, and Gao 2017; Hussain et al. 2017), stereochemistry (composition in d and l) (Makadia and Siegel 2011) or end-group functionalization (Wang et al. 2019).

Another important aspect is the choice of the preparation technique. There are a lot of procedures that can be used for the synthesis of nano and microparticles; they are summarized in Table 1.

**Table 1:** Different tecniques for PLGA MPs production

Techniques	References
Emulsion solvent evaporation–extraction method	Ospina-Villa et al. (2019), Grizić and Lamprecht (2020), Hernández-Giottonini et al. (2020)
Emulsification solvent diffusion method	Calderó et al. (2020)
Supercritical fluid emulsion	Cricchio et al. (2017)
Coacervation	Makadia and Siegel (2011)
Spray drying	Kim et al. (2019a, b)
Hydrogel template method	Wang et al. (2015)
Microfluidic systems	Amoyav et al. (2019)
Membrane extrusion emulsification	Zhang et al. (2020)
Particle replication in non-wetting templates (PRINT) technique	Perry et al. (2011)
Electro hydrodynamic atomization (EHDA) or electro-spraying	Jafari-Nodoushan et al. (2015)
Particles from gas saturated solutions (PGSS) method	Keles et al. (2014)

Depending on the production technique, MPs show specific size, polydispersity index (PDI), and morphological characteristics. These features are fundamental to ensure stability, encapsulation, and an adequate release of the drug (Busatto et al. 2018). For these reasons, preparation techniques are chosen according to the drug and to the specific application of the MPs (Bao et al. 2011; Wan and Yang 2016). For water-soluble molecules and highly instable molecules like proteins, the gold standard is represented by the double emulsion technique, because this procedure is able to protect proteins from degradation (Ma 2014); conversely, lipophilic molecule scan be encapsulated by single emulsion techniques (Wischke and Schwendeman 2008). All these features combined with the PLGA degradation characteristics, make PLGA MPs a very interesting system for drug delivery.

However, PLGA MPs have some negative aspects such as the tendency to aggregate to each other, the presence of organic solvent residues, high initial burst during the drug release or an incomplete release, and instability of encapsulated biomolecules. Some of these issues are usually overcome by adding different excipients to the formulations. Recently, new formulations to limit the amount of the initial burst and to obtain a delayed drug release have been developed. For example, it is possible to add sodium chloride (NaCl) or other additives to water or oil phase (Patel et al. 2012) or to encapsulate the drug in nanocrystals, which in turn are loaded in the MPs (Wang et al. 2017). Aggregation could be avoided by adding excipients to the formulations as lubricant agents such as magnesium stearate (O’Connor et al. 2019) and oleic acid (Cocks et al. 2015). Protein stabilizer, as divalent ions, ovalbumin and hydroxyl propyl-β-cyclodextrin,or other excipients, as trehalose or Tween, have been successfully used to improve protein and enzyme stabilities into PLGA MPs (Osman et al. 2013). Conversely, one of the most critical aspects is the employment of toxic reactive as organic solvents (i.e., dichloromethane or chloroform) (Table 2); consequently, there is a great interest in developing greener and safer procedures when preparing PLGA MPs (Sah 2000). Nowadays, many researchers proposed green solvents as valid alternative to the toxic organic ones, to improve final formulation safety but also the security during the MP preparation. In literature, there are reported non-toxic solvents as glycofurol, dimethyl carbonate, propylene carbonate (Table 3) or supercritical CO_2_ (Nunes and Duarte 2011). Moreover, the treatment of solid MPs with supercritical CO_2_ is proposed as a green procedure for generating pores onto particle surface (Koushik and Kompella 2004; Tran et al. 2013, 2012).

**Table 2:** Most common **s**olvents employed as oil phase for the preparation of PLGA MPs

Solvents	Non-halogenated	Halogenated	References
Dichloromethane		X	Çetin Altındal and Gümüşderelioğlu (2019), Cocks et al. (2015)
Chloroform		X	Wise (2000)
Tetrahydrofuran	X		Tang et al. (2012)
Hexafluoro-isopropanol		X	da Silva-Junior et al. (2009)
Ethanol	X		Yang et al. (2007)
Isosorbide dimethyl ether	X		Haji Mansor et al. (2018)
*N*-Methylpyrrolidone	X		Bilati et al. (2005)
Ethyl acetate	X		Gentile et al. (2016), Li et al. (2018)
DMSO	X		Choi and Park (2006)
Glycofurol	X		Allhenn and Lamprecht (2011)
Acetone	X		Herrmann (1995)
Methyl ethyl ketone	X		Sah et al. (1996)
Propylene carbonate	X		Grizić and Lamprecht (2018)
Ethyl propionate	X		Kang et al. (2014)
Ethyl formate	X		Sah (2000)
Dioxane	X		Wang et al. (2017)
Methyl chloride		X	Klose et al. (2006)
Dimethyl carbonate	X		Esposito et al. (2016)
Acetonitrile	X		Morales-Cruz et al. (2012), Rodriguez de Anda et al. (2019)

**Table 3:** Most common porogen agents employed for the preparation of porous PLGA MPs

Porogen agents	Type	References
NaCl	Osmogens	Patel et al. (2012)
Ammonium bicarbonate	Gas-foaming agents	Lee et al. (2010)
Pluronics	Extractable porogens	Kim et al. (2006); Ni et al. (2017)
Sodium oleate	Extractable porogens	Jiang et al. (2014)
Gelatin	Extractable porogens	Kim et al. (2010)
Mustard oil	Extractable porogens	Biswal et al. (2020)
BSA	Osmogens	Lee et al. (2010)
Cyclodextrins	Osmogens	Lee et al. (2007), Ungaro et al. (2009)
Mineral oil	Extractable porogens	Nasr et al. (2013)

In this review, we will discuss the recent developments in the preparation of PLGA MPs by emulsion techniques. We describe how the preparation method can be adapted to tailor the MPs properties and we focus on the encapsulation of different kind of drugs, the physiochemical characterization of MPs and their in vitro and in vivo applicability. Our aim is to highlight the future directions to address for the optimizing of porous PLGA MPs as drug delivery systems in biomedicine.

## PLGA MP production technique: emulsification–evaporation method

Emulsification–evaporation method is the easiest preparation method of PLGA MPs and involves the preparation of a formulation based on single or multiple emulsions combined with the evaporation of the organic solvent (Wise 2000). This technique can be carried out by batch process or more sophisticated methods such as membrane emulsification method (Ito and makino 2004) or microfluidic system (Brzeziński et al.^2019^; Microfluidic Systems 2020). This section will explain the basic principle and the recent advances of this process, including the batch procedure (single and double emulsion techniques) and the microfluidics technology.

### Single emulsion technique

**Fig. 2:**
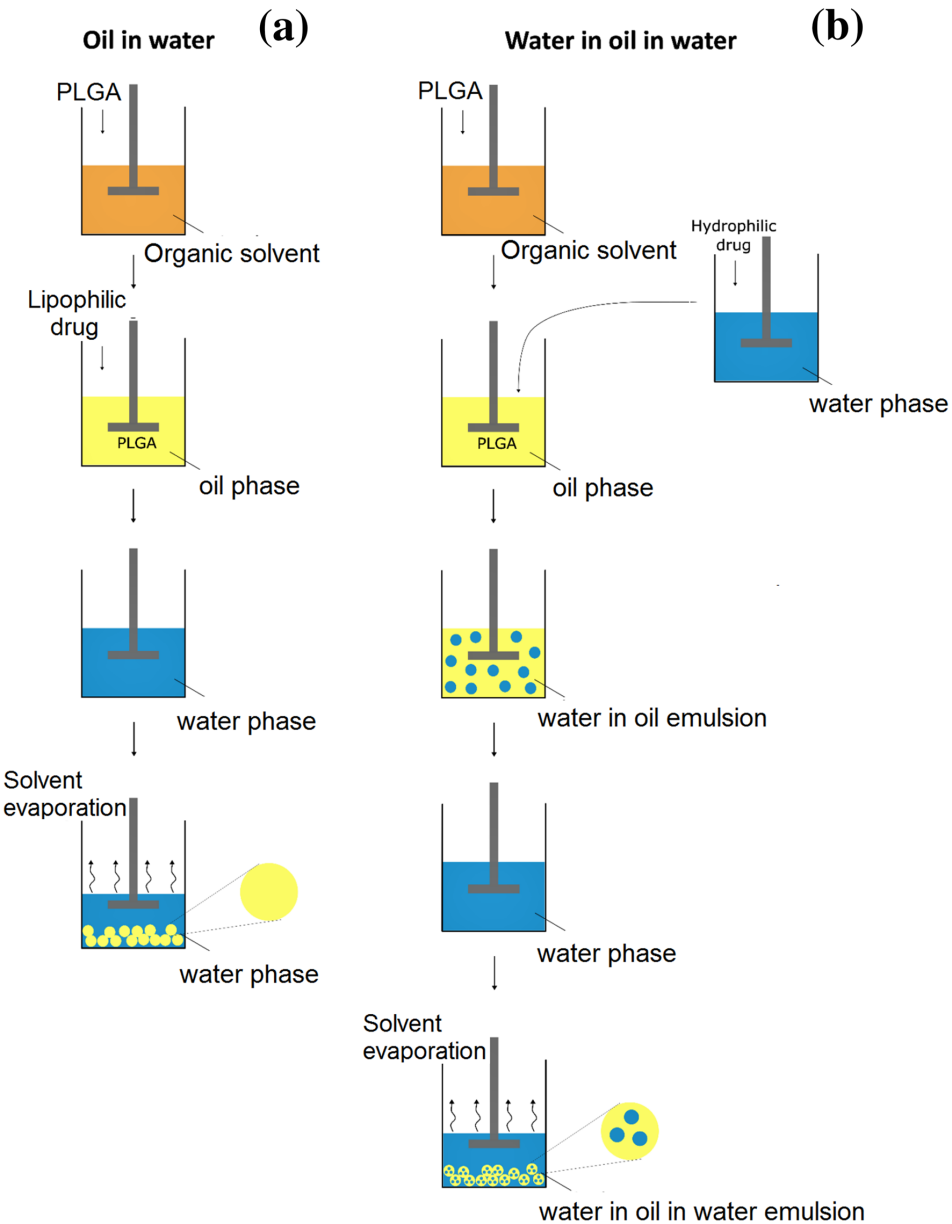
Schematic representation of emulsion solvent evaporation techniques for PLGA MPs production: O/W single emulsion method (**a**) and W/O/W double emulsion (**b**)

The single emulsion technique is one of the most common procedures used to encapsulate lipophilic molecules such as steroids [Budenoside (Oh et al. 2011) and Leuprolide (Park et al. 2019a, b)], nutraceuticals (curcumin) (Wischke and Schwendeman 2008), drugs [Donepezil (Vora et al. 2017) and Ketoprofen (Hasan et al. 2015)]. This technique involves the formation of an oil in water (O/W) emulsion (Wischke and Schwendeman 2008) in which polymer and drug are dissolved together in an appropriate solvent. At present, halogenated solvents with a low boiling point such as dichloromethane, chloroform, hexafluoro-isopropanol and/or non-halogenated solvents like ethyl acetate, isopropanol, methyl ethyl ketone, acetone and benzyl alcohol are preferred (Table 2), otherwise mixed solvent are used (Ni et al. 2017). This solution represents the oil phase (O) and it is added by sonication or homogenization to the water phase, that is made up of water and a surfactant or emulsifying agent as polyvinyl alcohol (PVA), polyethylene glycol sorbitan monolaurate (Tween), sorbitan monooleate (Span), sodium dodecyl sulfate (SDS) to form the final emulsion (Fig. 2a). The mature MPs are formed during the elimination of the solvent by evaporation that can be facilitated by a continuous stirring or using an under-pressure solvent draw system (Tan and Danquah 2012). Good results with this technique have been achieved in neurological disorders (Garbayo et al. 2012). It has been confirmed that in Parkinson disease (PD), neuron death is induced by oxidative stress as suggested by the increase in production of reactive oxygen species (ROS) and by the decreased activity of ROS-sensitive enzymes able to avoid the accumulation of neurotoxic compound into the brain such as lecithin–cholesterol acyltransferase (LCAT) (Di Natale et al. 2018). Fernandez et al., treated PD-model animals with rasagiline mesylate (RM), a potent anti-oxidant molecules, encapsulated within PLGA microspheres. Even if, non-statistically significant differences have been found between RM administration in solution or within microparticles, the possibility to control the release administration of RM every 2 weeks makes this innovative therapeutic system a fascinating approach for the treatment of PD (Fernández et al. 2012).

In the last years, it was registered a tendency of many authors in using non-toxic solvents such as dimethyl sulfoxide (DMSO), glycofurol and liquid PEG, propylene carbonate, ethyl propionate, dimethyl carbonate, ethyl formate; this choice is born from the need to develop safer preparations processes and to follow International Conference on Harmonization (ICH) guidelines for residual solvent (Q3C(R4)). ICH guidelines divided solvents in three classes (class I: solvent that should be avoided, class II solvent to be limited and class III with a low toxicity potential). Moreover, ICH guidelines indicated the permitted daily exposure (PDE), the concentration limit for each solvent and also the analytical method to estimate the amount of solvent residue (Committee for Human Medicinal Products 2018). Using a non-toxic solvent, it is possible to overcome these issues and to obtain a safer final product for pharmaceutical markets (Grizić and Lamprecht 2018).

PLGA MPs characteristics can be easily modified by adding excipients to the water or oil phase to improve porosity (Lee et al. 2010), the encapsulation efficacy (Ungaro et al. 2010), the control release and drug stability (Van De Weert et al. 2000). For instance, to increase porosity, porous agents such as pluronics (Kim et al. 2006), homogenized gelatin (Kim et al. 2010), cyclodextrinscan be used. Porogens can be divided in: osmosis-inducing agents, extractable porogens and gas-foaming agents depending on the pore formation mechanism involved (Table 3). Osmosis-inducing agents, or osmogens, produce pores owing to osmotic pressure differences between the internal and external phases (Lee et al. 2010). Extractable porogens generate pore structures because of the time difference between PLGA solidifying and porogen removal from oil phase to water phase (Kim et al. 2010), while pore formation mechanism of gas-foaming agents involves the development of gas bubbles (i.e., carbon dioxide, CO_2_) due to acidic solution or elevated temperature (Yang et al. 2009). Otherwise, it is possible to induce pore formation by treating solid MPs with supercritical CO_2_, which is able to soak in the PLGA matrix and expand to form pores upon isothermal depressurization (Koushik and Kompella 2004; Tran et al. 2013, 2012).

As previously reported, MP porosity modulation is a fundamental aspect in the control of the drug release kinetics. This topic will be described deeply in the following section. Single emulsion technique is a very simple method to produce PLGA MPs; however, this method shows some negative aspects such as low encapsulation efficacy for hydrophilic molecules that are better encapsulated using a double or a multiple emulsion technique.

### Double emulsion

The double emulsion technique is the main method to encapsulate water-soluble drugs such as proteins, peptides, and vaccines because of the simplicity of the process, low cost instrumentation and good control of the process parameters (Ansary et al. 2014). Differently to the single emulsion technique, this method involves the formation of multiple emulsion water in oil in water (W/O/W) that is a dispersive system consisting of three separated phases. The first W/O emulsion is obtained by adding an aqueous solution of hydrophilic drugs to the polymer solution that are then emulsified by a sonicator or a high-speed homogenizer; this first emulsion is called: “primary emulsion”. The primary emulsion is then added to a large volume of continuous water phase in presence of an emulsifier agent (PVA) to form the final W/O/W double emulsion, which is called “secondary emulsion”. To obtain micro- and nanoparticles solidification, the final step of the process involves solvent evaporation or extraction as for the single emulsion technique (Fig. 2b).

Usually this technique is used for the encapsulation of anti-viral agents, proteins (Formiga et al. 2010; Ospina-Villa et al. 2019) and nucleic acids (Salem 2008) that, however, suffer of high instability. For this reason, some stabilizing interface-active excipients such as sugars (trehalose, sorbitol) are added into the internal water phase solution that are able to protect proteins from aggregation as well as from denaturation during emulsification process (Han et al. 2016; Ma 2014). Recently, Azizi et al. (2020) applied this technique to produce W/O/W PLGA nanoparticles loading chondroitinase ABC (ChABC), a bacterial enzyme used to digest the chondroitin sulfate proteoglycans (CSPGs) to contrast the spinal cord injury (SCI).

Both single and double emulsion techniques used in solvent evaporation method give micro- and nanoparticles with an unregularly morphology, a high polydispersity and a low content of drug loaded. These negative aspects could be overcome using sophisticated procedures such as membrane emulsification method or microfluidic devices in which the critical points in the solvent evaporation method mechanical agitation, long time of solvent evaporation are eliminated.

### Microfluidics

The traditional solvent evaporation techniques (single and double emulsion methods) are widely used for the preparation of PLGA MPs because of the low costs and the simplicity of the procedures, but they are associated to low reproducibility, wide particle size distribution, less tunable morphology and low encapsulation efficiency. For these reasons, they have been recently replaced by alternative procedures able to produce, in a controlled way, MPs with the desired properties (He et al. 2019). In this scenario, one of the most efficient MPs production method is based on microfluidic technology.

**Fig. 3:**
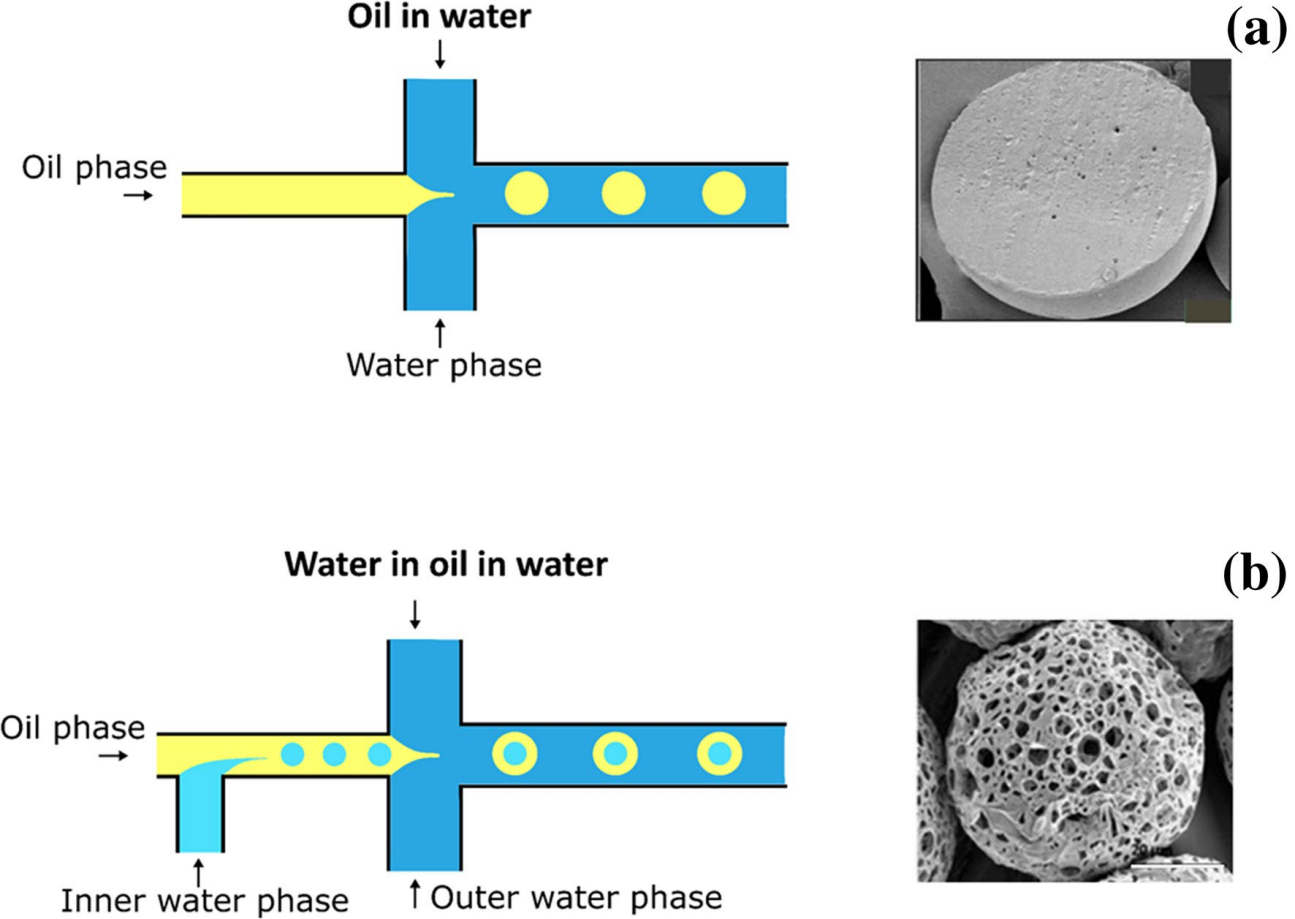
Schematic representations of microfluidics devices for preparation of PLGA MPs by O/W emulsion (**a**) and W/O/W emulsion (**b**) techniques. Adapted from Yanliang et al. (Fan et al. 2019) and Benzion et al. (Amoyav et al. 2019)

Microfluidic devices are made up of microchannels of various materials such as polymers (e.g., poly-dimethyl siloxane (PDMS), poly-(methyl methacrylate) (PMMA), polycarbonate (PC) and polyimide), metal (aluminum), phenol formaldehyde resin-based and glass capillaries (e.g., quartz, fused silica, and borosilicate). The basic principle is the same of the formation of an O/W emulsion (Fig. 3a) or multiple W/O/W emulsions (Fig. 3b), but the internal phase is pushed in the continuous one by a microchannel system to give a monodispersed emulsion (Chong et al. 2015). For the preparation of multiple emulsions, microchannels usually need selective surface treatment. Nowadays, different PDMS surface modification approaches have been used including covalent modification, glass coating, treatments with ionic wetting agent, oxidation, chemical vapor deposition, oxidation of the PDMS surface and layer-by-layer (LbL) deposition of positively and negatively charged species onto the surface. Montazeri et al. proposed a method to produce a partially hydrophilic/hydrophobic microfluidic device by adding a surfactant to PDMS solutions before curing (Chong et al. 2015; Montazeri et al. 2016). Particularly, they added to PDMS a super spreading biodegradable non-ionic surfactant, Silwet L-77^®^ that is able to improve the PDMS wettability by spreading on hydrophobic solid surfaces. Microfluidic chips were successfully used to prepare multidrug-loaded PLGA MPs (Rezvantalab and Keshavarz Moraveji 2019), encapsulating both hydrophilic and lipophilic compounds using the double emulsion method (Li et al. 2017). These devices can be divided in droplet-based (segmented) and continuous microfluidic devices. Droplet-based devices are preferred to produce MPs, while continuous devices are usually used for the preparation of nanoparticles (Rezvantalab and Keshavarz Moraveji 2019). The droplet-based system can be distinguished by the droplet breakup mechanism: cross-flow, co-flow, and flow focusing (Rezvantalab and Keshavarz Moraveji 2019; Vladisavljević et al. 2014). The main difference between these three devices is given by the geometry of the channels that affects the viscous shear force for droplet breakup as well as the final characteristics of the MPs. In cross-flow devices, the two phases come into contact in a junction with various angles such as T-junctions, Y-junction; double T-, V- and K-junction (Chong et al. 2015). The T-junction is the most common junction. Co-flow or coaxial junction dispersed phase channel flows in parallel into the aligned continuous phase channel, in other words the device is made up of two concentric channels. Flow focusing is similar to co-flow geometry but the size of the droplet is affected by capillary number and by the dispersed phase to continuous phase flow rate ratio. Anyway, many microfluidic devices are made up of combination of microchannels. For example, the liquid-driven co-flow focusing (LDCF) process has been developed to fabricate curcumin-loaded PLGA MPs. This chip is made up of three injection pumps, a core device including a coaxial needle and a pressure chamber, a collector, a safety waster system and a monitor system. This chip gives curcumin-loaded MPs with a narrow size distribution while maximizing drug encapsulation efficiency and drug loading rate with a programmed drug release profile (Dwivedi et al. 2018). Through microfluidics, it is possible to obtain MPs with a narrow size distribution, a precise control of the droplet size and a correct morphology (Amoyav et al. 2019). These characteristics are involved in drug release; the uniformity of the particles provides a precise control of the release kinetics and of the encapsulation efficiency. Particles with a narrow size degrade faster than the bigger ones, so a huge PDI is associate to an initial burst release and an uncontrolled drug release because of the presence of MPs of different size (He et al. 2019). Microfluidic devices give an excellent control of the whole preparation and manipulation processes: flow rate, pressure and viscosity can be modulated, and the fine control of these parameters eliminates errors and allows to achieve a process waste solution of almost 0% (Anon n.d.). Another advantage is the homogeneous mixing of the fluids and the possibility to work in the microliters since picolitres range (Dwivedi et al. 2018). Anyway, microfluidic technology shows some negative aspects as the compatibility of the polymers used for the microchannel production with various organic solvents. For example, PDMS or PMMA that are the most common materials used in the fabrication of microfluidic devices are susceptible to swelling when exposed to strong solvents such as acetone. Swelling of microfluidic channels affects fluid flow generating uncontrolled MPs in size and morphology.

### Physicochemical characterization: morphology, size, aerodynamic diameter, and zeta potential

The physicochemical properties of PLGA MPs are determined by several factors: production method (Busatto et al. 2018; Wang et al. 2015), processing and sterilization (Keles et al. 2015), kind of polymers (i.e., polymer functionalization, molecular weight, and monomer composition) (Beck-Broichsitter et al. 2016; Ni et al. 2017; Washington et al. 2018; Xia et al. 2017), drug (Capan et al. 2003), formulations parameters (Hernández-Giottonini et al. 2020), presence of excipients such as porogen agents(Oh et al. 2011), osmotic agents, stabilizers, surfactants (Li et al. 2018).

Morphological analysis can be done with electron microscopy (scanning electron microscope SEM, field emission scanning electron microscopy, FESEM) (Ding et al. 2018) to extract geometric diameter (*d*_g_), pore diameter (*d*_p_) and pore number and by confocal microscopy (CF) to see the internal structure of the pores. The particle size can be also obtained by optical microscope (OM) and light laser diffraction.

Morphological features, size, and PDI are important factors in the control of the kinetics of drug release. In literature, there are many mathematical models able to predict the kinetics of release of a drug through the evaluation of morphological and dimensional parameters of particles (Busatto et al. 2018; Ford Versypt et al. 2013). These aspects will be better explored in the next section. Generally, homopolymer PLGA MPs produced by emulsification method show spherical shape with smooth surface (Wang et al. 2015) and a negative value of *z* (ζ) potential due to the presence of carboxyl groups at the end of polymer chains. When PLGA is functionalized with PEG, the presence of PEG produces a less negative value of *ζ*-potential due to the neutralization of PLGA negative charges (Patel et al. 2012). The functionalization of PLGA with PEG is often carried out to reduce polymer hydrophobicity and improve the encapsulation of hydrophilic substances (Patel et al. 2012). In the emulsion solvent evaporation method, the morphology of MPs is influenced by the rate of polymer precipitation at the solvent removal step: a slow elimination of the organic solvent gives smooth surfaces, generated by a slow precipitation of the polymer; instead, a quick removal produces a porous surface. Using ethylacetate as organic solvent of the oil phase, PLGA MPs express a rough surface because its high boiling temperature does not allow a complete solidification process (Li et al. 2018).

Pore size can be modulated choosing the appropriate solvent: solvent with a lower volatility gives rise to large pores, while solvent with a higher volatility to smaller pores (Wang et al. 2019). Also the concentration of polymer influences MPs porosity: increasing polymer concentration from 1 to 3% and 5%, reduces the porosity degree of the particles from 83 to 71% and 64%, respectively, without conferring a statistically significant difference in the mean diameter of the MPs (Amoyav et al. 2019). MPs porosity is fundamental for drug delivery: porous particles have a broader surface area and a lower density that allow to get a faster release of the drug (Busatto et al. 2018; Kim et al. 2017). Usually, the addition of porogen agents to the formulation increases MPs porosity and an appropriate porogen agent can be selected based on the kind of application, drug compatibility and the preparation technique. One of the most commonly used is ammonium bicarbonate (as gas forming agent) (Oh et al. 2011); however, there are several other substances including osmolytes (Lee et al. 2010) as salts (NaCl), phosphate buffer solution (PBS), protein (albumin) and extractable porogens as polymers (pluronic) and fatty acid salts (Table 3).

In literature, many unconventional porogen agents are reported, among these, PVA shows very interesting properties. Particularly, Ni et al., used PVP as a new extractable porogen agent, combined with the single emulsion (O/W) solvent evaporation method to prepare large porous Cinaciguat-loaded PLGA MPs for the treatment of pulmonary hypertension via inhalation. PVA not only simplifies the procedure but increase the encapsulation of hydrophobic drugs (Ni et al. 2017). The same group demonstrated also that the end group of PLGA influences the porosity and the size of each pore (Ni et al. 2017). They described a “one-step” emulsification technique for the preparation of porous MPs in which porosity was induced by a spontaneous formation of W/O emulsion droplets in the organic solvent using polymeric surfactant (polymeric PEG-PLAs) instead of non-polymeric surfactant (i.e., Tween). The presence of PEG-PLAs also increased *d*_g_ and *d*_p_ and***, ***moreover, this increase was proportional to the molecular weight of the PEG block in the polymeric surfactant (Nishimura et al. 2017; Takami and Murakami 2014).

The MPs size is related to the type of surfactants and their concentration. Novedori et al., compared four types of surfactant PVA, Tween 20, Span 20 and SDS to study their effect on MPs. They demonstrated that PVA could produce the smallest MPs dimensions, while SDS the biggest one. In particular from this comparative study, PVA showed to give the appropriate size value for the preparation for MPs for inhalatory route (Noviendri et al. 2016). Particle size is of fundamental importance for the chosen route of administration, for example, for intra-myocardial applications, the appropriate size value is around 5–8 um while for inhalatory or parental is around 1–5 μm. Recently, Hernández-Giottonini et al., evaluted the importance of fomulation composition both using single emulsion and nanoprecipitation tecniques to obtain uniform PLGA nanoparticles. They analyzed the role of the polymer and surfactant concentrations, the organic solvent fraction and the sonication amplitude (single emulsion technique) and the injection speed (nanoprecipitation) effects; moreover, they evaluated the influence of purification method and cryoprotectants. As reported, the polymer composition and concentration affect the physicochemical characteristics (size, PDI, and zeta potential) while the organic solvent phase mostly controlled the particle dimensions in single emulsions technique while PLGA and PVA concentration influenced in the nanoprecipitation one (Hernández-Giottonini et al. 2020).

Other important parameters, for the administration of MPs by inhalatory route, are mass median aerodynamic diameter (MMAD), geometric standard deviation (GSD) (Nishimura et al. 2017; Patel et al. 2012). For the calculation of theoretical MMAD, it is important to know the tapped density, while the aerodynamic diameters can be easily determinate by cascade impactor (CI) or Aerodynamic particle sizer (APS). Moreover, CI can be used to evaluate the particle deposition ratio on the respiratory area (nasal cavity, pharynx, trachea, bronchi, and alveoli) (Takeuchi et al. 2018)**.** The aerodynamic performance is mostly influenced by the porosity of PLGA MPs. Nishimura et al., demonstrated the different aerodynamic behaviour of porous and not-porous particles: porous particles deposits predominately in the nasal cavity or bronchi, a little in the trachea and pharynx, whereas non-porous particles deposit in the nasal cavity (Nishimura et al. 2017). The tapped density can be determined by mechanically tapping a known weight of the particles in a 5 mL-graduated cylinder. It is expressed as the ratio between the sample weight and the volume of the particles occupied after 1250 tappings (Gupta et al. 2011; Nishimura et al. 2017; Ungaro et al. 2009). This parameter gives information about the ability to reach the deepest sections of the respiratory system.

All morphological and chemical–physical characteristics of PLGA MPs described in this section and resumed in the Table 4 have a pivotal role in determining the release kinetics of the drug: the formulator must then select accurately the materials of use and preparation methods to ensure the desired release of the drug. For example*,* Ospina-Villa et al., have encapsulated proteins from *Leishmania panamens* is into PLGA MPs by single emulsion technique, dissolving the protein in the oil phase made of DCM and PLGA. Particularly, they encapsulated two different kinds of protein from *L. panamensis*: LpanUA.22.1260, an insoluble protein over expressed in the *Leishmania promastigotes*, and LpanUA.27.1860, a soluble protein with a similar expression in the Leishmania amastigote and promastigote stages. The PLGA MPs had a good morphology and a sustained protein release for 10 days without degradation (Ospina-Villa et al. 2019). However, some proteins are more sensible and need more sophisticated formulation systems for their stability. Usually, to increase the protein stability, stabilizers such as bovine serum albumin (BSA), succinylated gelatin or PEG are added to the water phase where protein solution is solubilized, to avoid protein degradation during the preparation process (Hajavi et al. 2018; Panyam et al. 2003; Van De Weert et al. 2000). De Alteriis et al., successfully used BSA as protein stabilizer for vascular endothelial growth factor (VEGF) into PLGA MPs obtained by stamp-based method at room temperature exploiting solvent/non-solvent plasticization. This formulation showed a prolonged release of VEGF in active form PLGA MPs (De Alteriis et al. 2015).

**Table 4:** MP properties and the related techniques

Properties	Techniques	References
Shape	OM, CM, SEM	Ali et al. (2019), Nishimura et al. (2017)
Size	CM, SEM, OM, light laser diffraction	Amoyav et al. (2019)
PDI	Light laser diffraction	Gupta et al. (2011)
Porosity	SEM, OM	Amoyav et al. (2019)
ζ-potential	DLS	Ali et al. (2019), Costabile et al. (2018), Cricchio et al. (2017)
Aerodynamic diameter	CI, APS	Cocks et al. (2014), Nishimura et al. (2017)

## Drug encapsulation and in vitro and in vivo release

### Drug encapsulation and its loading efficiency evaluation

Many parameters could affect the drug loading in PLGA MPs, so it is fundamental to know which factors could be modified to improve the drug loading. Among these, PLGA features (such as MW, surface functionalization) are between the most studied factors. In particular, PLGA functionalization is related to drug loading and the end groups have a significant effect on drug encapsulation: Wang et al., studied the role of the end-group capping of PLGA for doxycycline hyclate encapsulation. They demonstrated that the acid end group gives lower encapsulation efficiency and faster doxycycline hyclaterelease rate, while a higher encapsulation efficiency was obtained for the ester-capped (Wang et al. 2019). Furthermore, it was shown that the nature of drug/PLGA interactions has a pivotal role in the molecule encapsulation; in particular, ionic interactions between the drug and polymer will result in higher incorporation in the non-end-capped polymers while if the interaction shows hydrophobic features, the end-capped polymers will show a greater incorporation. Moreover, different degradation rates can be reached using capped PLGA with similar molecular weights but bearing different end groups, for example, PLGA with an ethyl capping group showed a slightly faster degradation rate than the one with the hexyl group (Samadi et al. 2013).

As to proteins and peptides, the most used encapsulation technique is the double emulsion solvent evaporation. This technique is used for example to encapsulate nucleic acids in PLGA MPs. These particles, within size range 2–3 µm, can release the drug content into macrophages by joining the phagocytic process of these cells as a mechanism for uptake. However, these substances have a low loading efficacy that can be overcome using cationic excipients as the lipid 1,2-dioleoyl-3-trimethylammonium-propane (DOTAP), which is able to improve the drug solubility (Ungaro et al. 2010).

PLGA MPs can also be used in food industry to encapsulate anti-microbic agents as thymol (Zhu et al. 2019). This molecule shows broad-spectrum antimicrobial properties, but its volatile characteristics and strong odor represent a big limitation for the use in food products that could be easily overcome by microencapsulation. Zhun et al., prepared thymol-loaded PLGA MPs with an initial fast release followed by a slow and sustained release. These MPs demonstrated to have astrong antibacterial activity against Escherichia coli and Staphylococcus aureus confirmed by analytical tests in milk samples (Zhu et al. 2019). These results showed that thymol-loaded PLGA MPs could be used as antimicrobial and preservation additives in food industry (Zhu et al. 2019). Same molecules with a high water solubility such as gentamicin sulfate can be encapsulated by post-loading procedure by simply putting PLGA MPs into drug solutions. Post-loading eliminates the loss of drug during the washing process.

To know the amount of loaded drug and the loading efficacy (encapsulation efficacy, EE), it is necessary to break up the MPs formulation and evaluate the amount of drug through different techniques that are chosen based on the drug features. For example, the quantification can be done by spectrophotometric method (UV–vis) but also by high-performance liquid chromatography (HPLC), gel permeation chromatography (GPC), fluorometric assay, or ELISA test. The evaluation of the encapsulation capacity can be also done indirectly by measuring the drug content in the supernatant recovered after washing the MPs formulations.

Drug encapsulation efficiency is expressed as the percentage of drug in the fabricated particles with respect to the initial amount of drug used for loading.

Other methods/analyses have been recently reported in literature to evaluate encapsulation efficacy and structural and chemical integrity of drugs. For example, thermogravimetry analysis (TGA), glass transition (*T*_g_) temperature measured using differential scanning calorimetry (DSC) (Dwivedi et al. 2018), attenuated total reflectance-Fourier transform infrared (ATR–FTIR) (Noviendri et al. 2016) and X-ray diffraction (XRD) (Xia et al. 2017) of PLGA MPs were employed for controlling drug stability. The interaction between drug and polymer can be studied by FITR spectroscopy: Keles et al. (2014) used FTIR spectroscopic imaging to evaluate the redistribution and release of hGH from a range of PLGA/PLA MPs.

### Drug targeting

Another major challenge in the development of drug delivery systems is the drug targeting mechanism. Micro and nanocarriers can be able to achieve tissue or cell targeting through an active or passive mechanism (Singhvi et al. 2020). In passive targeting, the drug can be delivered to the target passively based on the longevity of the pharmaceutical into the site of interest; while the active targeting is based on the attachment of a specific recognition element like ligand receptor, antigen–antibody to the surface of the carriers to deliver drugs to a specific location (Behera and Padhi 2020). Poor tumour-targeting ability is the major impediment in the field of cancer treatments (Maeda and Khatami 2018). Many studies have shown that PLGA carriers are capable of providing tumour tissue-targeting modifying their surfaces with other functional materials which are specifically recognized by receptors on the specific cells resulting in the increased penetration of tumour cells via receptor-mediated endocytosis (Kamaly et al. 2012). Recently, PLGA nanoparticles (NPs) carrying multiple ligands have been investigated to maximize tumour-targeting efficiency. These PLGA NPs can passively target delivery to tumours through enhanced permeability and retention (EPR) effect, and actively target delivery to tumours based on ligand–receptor interactions modifying them for example with cell-penetration peptides (Fenaroli et al. 2018; Fotticchia et al. 2017). A classic example are paclitaxel (PTX)-loaded PLGA NPs, in which through a PEG linker a small molecule ligand of folate receptor was attached to the surface resulting in an increased reduction of tumour volume in HEC-1A tumour-xenograft mice compared with basic NPs (Liang et al. 2011). Another substantial improvement was also achieved exploiting the use of a prototypic tumour-penetrating peptides known as iNGR; (CRNGRGPDC) able to increase the tumour vascular permeability through interaction with the neuropilin (NRP). In this case, an increased survival of 1.6-fold was found in PTX-loaded iNGR-PEG-PLGA NPs treated mice with respect to unmodified NPs (Ruoslahti 2017). The targeting moieties can be also attached via an indirect method. The most studied approaches were based on the charge–charge interaction between the targeting moiety and the NP surface or using the strong binding affinity between avidin and biotin (Webster et al. 2013). In this context, Wu et al. coated the vitamin E-oligo(methyl diglycol l-glutamate) positively charged surface of PLGA NPs with the anionic hyaluronic acid; theHA-coated PLGA (HPLGA) NPs loaded with Docetaxel (DTX) showed a prolonged survival of 1.4-fold of A549-Luc lung xenograft mice compared to the free ones (Wu et al. 2017). As to biotin/streptavidin strategy, Cook et al., showed that biotin-conjugated RVG29 (fragment of the rabies virus coat protein) was positively attached to avidin-camptothecin (CPT)-loaded PLGA NPs, but no significant differences were found in median survival compared with control NPs in mice bearing intracranial glioblastoma (Cook et al. 2015). As for microcarriers, few data are available to date but very recently, Park et al., showed as the PLGA-based micro-sized discoidal polymeric particles (DPPs) can be used as lung-targeted drug delivery carriers. These DPPs were synthesized using a top-down fabrication approach and they were radio-labelled with the positron-emitting radionuclide ^89^Zr. Bio-distribution and PET images demonstrated that the DPPs significantly accumulate in the lungs degrading within a week. Moreover, in vivo cytotoxicity studies showed no significant toxicity. These findings suggested that these PLGA-based DPPs may be useful for the specific treatment of various lung diseases such as asthma or chronic and fibrosis pulmonary illnesses (Park et al. 2019a, b). Another recent approach is based on polymeric microneedle arrays. In this work, biodegradable bilayer MN arrays containing nano/microparticles are fabricated for a targeted and sustained intradermal drug delivery. A model drug as vitamin D3 (VD3) was loaded into PLGA nano/microparticles (NMP) throughthe single emulsion solvent evaporation approach. The particle size was from 300 nm to 3.5 μm and it was retained also after moulding of bilayer MN arrays. Ex vivo intradermal neonatal porcine skin penetration was quantitatively analysed revealing that the 74.2 ± 9.18% of VD3 was intradermally delivered. This proposed system could represent a promising strategy for a controlled transdermal delivery or a targeted intradermal administration (Vora et al. 2017).

### Drug release

#### In vitro

Several mechanisms rule drug release process from PLGA MPs: polymer degradation, water absorption, drug diffusion into PLGA matrix, erosion, and hydrolysis.

The degradation process of the polymer regulates the drug release from PLGA MPs so it is important to understand this mechanism and by which parameters it is affected. As previously reported, the mechanism of degradation involves the hydrolysis of ester linkages in the polymer backbone subsequent to water uptake. PLGA MPs degradation is affected by polymer characteristic as MW (Guo et al. 2015), composition monomer (lactic acid/glycolic acid ratio) (Washington et al. 2018), degree of crystallinity (Takeuchi et al. 2017), porosity (Klose et al. 2006) and end-group functionalization (Wang et al. 2019). Generally, a lower MW is associated to a faster degradation time mostly driven by water diffusion into the particle, while for PLGA with a high MW the process is a combination of diffusion and erosion that needs more time for matrix degradation.

Many studies reported that one of the most important characteristics that influence the degradation rate of PLGA is the monomer composition. Usually, an increase in glycolic acid percentage in the oligomers accelerates the weight loss of polymer: PLGA 50:50 (PLA/PGA) exhibited a faster degradation than PLGA 65:35, 75:25, and 85:15 because of the degradation of PGA proportion having a higher hydrophilicity (Makadia and Siegel 2011).

The internal structure of the particles, checked in terms of MW, pore formation and *T*_g_, is nowadays considered of primary importance in drug kinetics release (Mylonaki et al. 2018). On the other hand, considering that drug release is related to drug–polymer interaction, drug characteristics as MW, log-P, concentration also appear to be crucial (Mao et al. 2007). Usually, for hydrophilic molecules the release is associated to porous route, while the hydrophobic drugs cross the hydrophobic PLGA matrix.

In vitro drug release studies are carried out by suspending a specific amount of drug-loaded particles in an appropriate solvent, shaking at 37 °C to have a sink condition. At predetermined time points, a certain volume of sample is withdrawn after MPs sedimentation using centrifugation. The supernatant is collected for analysis while, the pellet is re-suspended in the same volume of fresh solvent.

The collected data can be fitted to various mathematical models such as zero order, first order, Higuchi, Korsmeyer–Peppas Weibull, Hixson–Crowell, Weibull, Hopfenberg, Gompertz and regression models (Casalini et al. 2014). Several studies of release kineticswith more sophisticated system asdialysis bag diffusion technique, flow-through cells, Franz cells and Enhancer cells are reported as valid release testing methods (Li et al. 2018; Patel et al. 2015; Tomic et al. 2016). Tomicet al., designed a very fast release process for a cyclic somatostatin analogue (MW ≈ 1 kDa)-loaded PLGA MPs based on flow-through method: they selected an accelerated condition (temperature release medium) for the in vitro drug release. In this study, the role of test condition was evaluated by chancing the pH, temperature, osmolality, and ionic strengthof the medium to define those that gave accelerated drug release without alteration of the entire mechanism. They used aphosphate buffer saline (PBS) as dissolution medium and evaluated each parameter separately. Especially, PBS solution was analyzed by varying pH (to 2 from 4 using ortho-phosphoric acid), temperature (45 °C and 40 °C), osmolality and ionic strength (50, 200, 380 m Osm/kg adjusted with NaCl or Glucose). A significant acceleration of the drug release was achieved using a solution of PBS 0.02 M at pH 2 and 45 °C. Particularly, the drug release was not significantly modified by varying pH value and temperature, while it was influenced by the composition of the test medium since the osmotic effect controls the erosions and the diffusion processes (Tomic et al. 2016).

The drug release could be generically divided in three steps: (i) initial burst release, (ii) lag time and (iii) final erosion-accelerated release (Wang et al. 2017). The initial burst release is linked to weakly bound or adsorbed molecules to the surface of MPs. It is associated to a loss of drug content, not desirable for a constant drug release; moreover, the initial high concentration of drug is often toxic. This initial phase can be avoided by several strategies; first of all, it is possible to take some precautions during the preparation method, as reducing the solvent evaporation time or choosing the right formulation component (Yeo and Park 2004). Drug release is highly affected by the porosity of PLGA MPs, and in particular by the concentration of the porogen used into formulations. Klose et al., evaluated the role of porosity in the drug release by taking as model drug Lidocaine by comparing porous and non-porous PLGA MPs. Particularly, the porous presence does not only offer a higher contact surface area, but also increases the mobility of the drug molecules and alters the contributions of the involved physicochemical processes. From this study, they asserted that the presence of the pores increased the mobility of the involved species modifying the drug release mechanism (Klose et al. 2006). Highly porous PLGA MPs provide fast and approximately complete drug release (Shiehzadeh et al. 2019): usually the initial burst release occurs in 30 min (Jiang et al. 2014). Especially, when the porogen agent is an osmogen, the drug content is leached out through the pore canals. However, a faster release is not always desired, so there are many strategies to obtain a sustained release. Among these, fatty acid conjugates of drugs are well known to prolong half-lives by promoting albumin binding (Kim et al. 2011). The initial burst release can also be avoided by formulating a composite microparticle (nanoparticle or cyclodextrin within PLGA MPs) (Dong et al. 2019). Successful results were obtained using cyclodextrin and/or nanoparticles for the delivery of insulin. This could be explained by the slower mobility of the drugs through the polymeric matrix (Hasan et al. 2015; De Rosa et al. 2005). The initial burst release is followed by the lag time. Usually, a long lag time is not desirable for instable drug, as protein or nucleic acid (Wang et al. 2015). In these cases, the elimination of this phase could be reached using more hydrophilic forms of PLGA, like copolymers of PLGA-PEG (Tran et al. 2011). A more controlled release can be achieved using a pH sensible polymer. In particular Li et al., used functionalized PLGA (with chitosan, mannose) to improve the nasal delivery of hepatitis B surface Antigen (HBsAg). They proved that PLGA functionalized MPs could cause a rapid antigen release at pH 5.0 and pH 6.0, but at the same time, a slow release at pH 7.4 (Li et al. 2016). PLGA MPs could be used for transdermal drug delivery of small biomolecules as a powerful substitute to subcutaneous or intravenous injections (Jain et al. 2017). To enhance skin permeability, different strategies have been used such as laser, thermal, or radiofrequency ablation or electrically assisted enhancement techniques (i.e., electroporation and iontophoresis) (Alkilani et al. 2015). However, these methods are related to several negative aspects such as skin damages and lower patient compliance (Jain et al. 2017). Microneedle patches containing nano- or microparticles are becoming a very useful system to overcome those problems since they are capable to penetrate skin stratum corneum avoiding the stimulation of the nerve endings; in other words it isa painless system for controlled and targeted transdermal drug delivery (Jamaledin et al. 2020a, b; Larrañeta et al. 2016). Vora et al., proposed a microneedle array with PLGA MPs, where microparticles were concentrated into needle tips by a simple centrifugation technique. In particular, they demonstrated that in vitro release of Vitamin D_3_ exhibited a typical tri-phasic release profile up to 5 days; they also investigated the role of mannitol, an osmotic agent giving a less porous structure, in the release. As the burst release involves a diffusion mechanism, addition of mannitol gives lower diffusion-based burst release due to the less porous structure of the system (Vora et al. 2017). Battisti et al., fabricated biodegradable polymeric microneedles for controlled intradermal drug release by a novel stamp-based method. Microneedles were made of a PVP fast dissolvable tip and a PLGA MPs body. After having loaded both the tip and the body with a model therapeutics, POXA1b lac case from Pleurotus ostreatus, a commercial enzyme used for the whitening of skin spots, microneedles action, and their indentation ability was assessed in an advanced in vitro skin model, showing their ability to control the kinetic release of the encapsulated compound (Battisti et al. 2019).

Many researchers proposed mathematical models as a useful tool to study drug formulations or to predict the release kinetics of molecules (Jakhmola et al. 2019; Peppas and Narasimhan 2014). Modelling drug release from PLGA MPs is important to predict and study in vitro and in vivo releases to obtain a fine control on the drug release kinetics, and so to optimally design drug formulations. In silico models give a more deeply comprehension of the physics and chemistry of the drug release process, decreasing also the number of experiments. Particularly, many models are based on the degradation of drug-loaded PLGA MPs and the consequent drug release process from such devices (Casalini et al. 2014)*.* Recently, a mathematical model able to predict the progesterone (Busatto et al. 2018) and florfenicol (Karp et al. 2019) releases from PLGA MPs of different size and different PLGA MWs was realized, by taking into account the drug dissolution and diffusion in the polymeric matrix and the autocatalytic effect of PLGA degradation. In particular, considering that the main mechanism of mass transport was the diffusion of the drug through the polymeric matrix, the release from PLGA MPs was described by a model based on Fick’s second law of diffusion Eq. (1):

1 $$ \partial C_{d} /\partial t = (1/r^{2} )(\partial /\partial r)\left( {r^{2} D_{d} \partial C_{r} /\partial r} \right) + S\quad t < t^{*} , $$

where ${C}_{d}$ is the concentration of drug dissolved in the polymeric matrix, ${D}_{d}$ is the effective diffusion coefficient of the drug in the polymeric matrix, $r$ is the radius of the MPs and $S$ is the source term. ${t}^{*}$ is the time at which the mass transfer in the polymer starts being not the preferential transport mechanism during the release process, since, as degradation proceeds, the morphological changes can influence the transport mechanism. This model showed to be in agreement with experimental results. Amini-Fazl and Mobedi (2020) proposed a theoretical model based on diffusion-control release together with semi-empirical models, capable of calculating the release rate constants, release exponent and average drug diffusion coefficients and to evaluate their variations with PLGA MW. In detail, the authors included Ritger–Peppas, Weibull and Peppas–Sahlin formulas and a theoretical one obtained from Fick’s second law. Ritger–Peppas or power-low model (Ritger and Peppas 1987) for drug release is represented by Eq. (2):

2 $$F={k}_{RP}{t}^{n},$$

where $n$ is the release exponent indicating the drug release mechanism and ${k}_{RP}$ is the release constant incorporating structural and geometrical characteristics of the drug form.

The Weibull model (Dash et al. 2010) is represented by Eq. (3):

3 $$F=100 \left(1-\mathrm{exp}\left(-{t}^{\beta }/\alpha \right)\right),$$

where $\alpha$ defines the time scale of the process and $\beta$ the release profile curve.For $\beta$=1 the curve is exponential, for $\beta >1$ and for $\beta <1$ the curve is parabolic.

Peppas and Sahlin (Ritger and Peppas 1987) diffusion-relaxation model is composed by Fickian and non-Fickian transport terms Eq. (4):

4 $$F={k}_{1}{t}^{m}+{k}_{2}{t}^{2m}$$

where, ${k}_{1}$ and ${k}_{2}$ are constants related to Fickian and non-Fickian kinetics, respectively, and $m$ is the diffusional exponent for a device of any geometric shape which inhibits controlled release.

The solution of the theoretical model based on Fick’s law for a solute from spherical boundary conditions gives Eq. (5) (Murzin and Heikkilä 2014):

5 $$F=1-6/\pi \sum_{n=0}^{\infty }exp\left(-D{n}^{2}{\pi }^{2}t/{r}^{2}\right)/{n}^{2}$$

where, $\mathrm{D}$ is the diffusion coefficient and $\mathrm{r}$ is the radius of microspheres.

Di Natale et al. (2020) suggested a tuneable release of curcumin using a combination of different PLGA MPs supported by an in silico non-linear first-order model cable of predicting and obtaining intermediate situations to guarantee prolonged or fast drug release, avoiding the necessity of realizing further experiments. This approach can be applied to other molecules besides curcumin, so that the release of drugs will happen in a more controlled amount with a better timing, optimizing the therapeutic efficiency and, therefore, decreasing possible side effects.

#### In vivo

As previously, described, biocompatible and biodegradable PLGA polymers have been selected as outstanding delivery carriers for controlled in vivo release of drugs, peptides, and proteins. Here we report some novel applications of PLGA MPs in different pathologies.

Recently, Kim et al. (2019a, b) described a formulation composed by bupivacaine-loaded PLGA MPs (BPC-PLGA-MPs) and fibrin glue (FG), to reduce and control postoperative pain. Currently, the patients affected by post-surgical pain are treated with long-term oral and/or patch treatments, which often cause systemic toxicity. To solve this problem and to create a sustainable drug release, bupivacaine was loaded in PLGA MPs, prepared with the O/W emulsion, and subsequently, the drug-loaded MPs were inserted into fibrin glue to create an additional diffusion barrier able to reduce the initial burst and to control the drug release. In vivo experiments were conducted in animals with neuropathic pain caused by spinal nerve ligation. Interestingly, only the animals treated with FG_BPC-PLGA-MPs but not with control formulations, showed an intense increase in paw withdrawal threshold (PWT) during the entire testing period (10 days) due to longer drug release. Considering that the actual approved formulation of bupivacaine is active for only 72 h, this approach could eliminate multiple daily injections of bupivacaine improving patient compliance (Kim et al. 2019a, b).

Another research group investigated the potential application of PLGA MPs in tuberculosis (TB). This pathology is still one of the major causes of death in the world, affecting both adults and children. The inhalation of infected droplets causes contagion and the subsequent navigation of the airways leads to immune activation and onset of the disease. Currently, an interesting approach against TB is to stimulate the immune system in favor of the host in a strategy called host-direct therapy (HDT) (O’Connor et al. 2019). Vitamins are valid HDT candidates and, in particular, the active metabolite of vitamin A, ATRA, showed immunomodulatory effects and it was demonstrated able to reduce the bacterial loading. Unfortunately, vitamin A is poorly soluble in aqueous buffers and instable during production and storage due to its sensitivity to light, heat, and oxygen. With the intention to overcome these obstacles and create a controlled release ATRA treatment for TB suitable for inhalation, using spray-drying technique, ATRA-loaded PLGA MPs (ATRA-PLGA-MPs), with inhalable size, were developed. In vivo studies were performed in an acute model of Mycobacterium tuberculosis (Mtb) infection BALB/c mice infected intranasally with H37Rv. After only three doses of ATRA-PLGA-MPs, intratracheally injected, the bacterial load in the lungs was significantly reduced (O’Connor et al. 2019).

PLGA MPs were also applied for the treatment of several infectious diseases for humans and animals. Particularly, Vilos and co-workers developed new PLGA MPs loaded with ceftiofur (cef-PLGA), a third-generation cephalosporin used exclusively in animals for the cure of respiratory disease. PLGA MPs were produced by the double emulsion W/O/W method. The in vivo efficacy of cef-PLGA was investigated in rats infected with *Salmonella typhimurium*. The animals were divided into three groups: injected with (i) saline, (ii) non-encapsulated ceftiofur and (iii) cef-PLGA. The injection was performed 2 days before the inoculation of *S.* *typhimurium* and the effects were evaluated 6 days after the *S. typhimurium* injection. To evaluate the efficiency of cef-PLGA, the quantification of bacteria (CFU/mg of tissue and feces) at the end of the experiment in spleen, liver, intestine, and colon samples was performed. Microparticles demonstrated a better antibacterial effect than the free ceftiofur upon infection, reducing systemic disease. Indeed, the animals treated with non-encapsulated ceftiofur showed *S. typhimurium* in intestine and colon while the animals injected with cef-PLGA showed bacteria only in the colon.

**Fig. 4:**
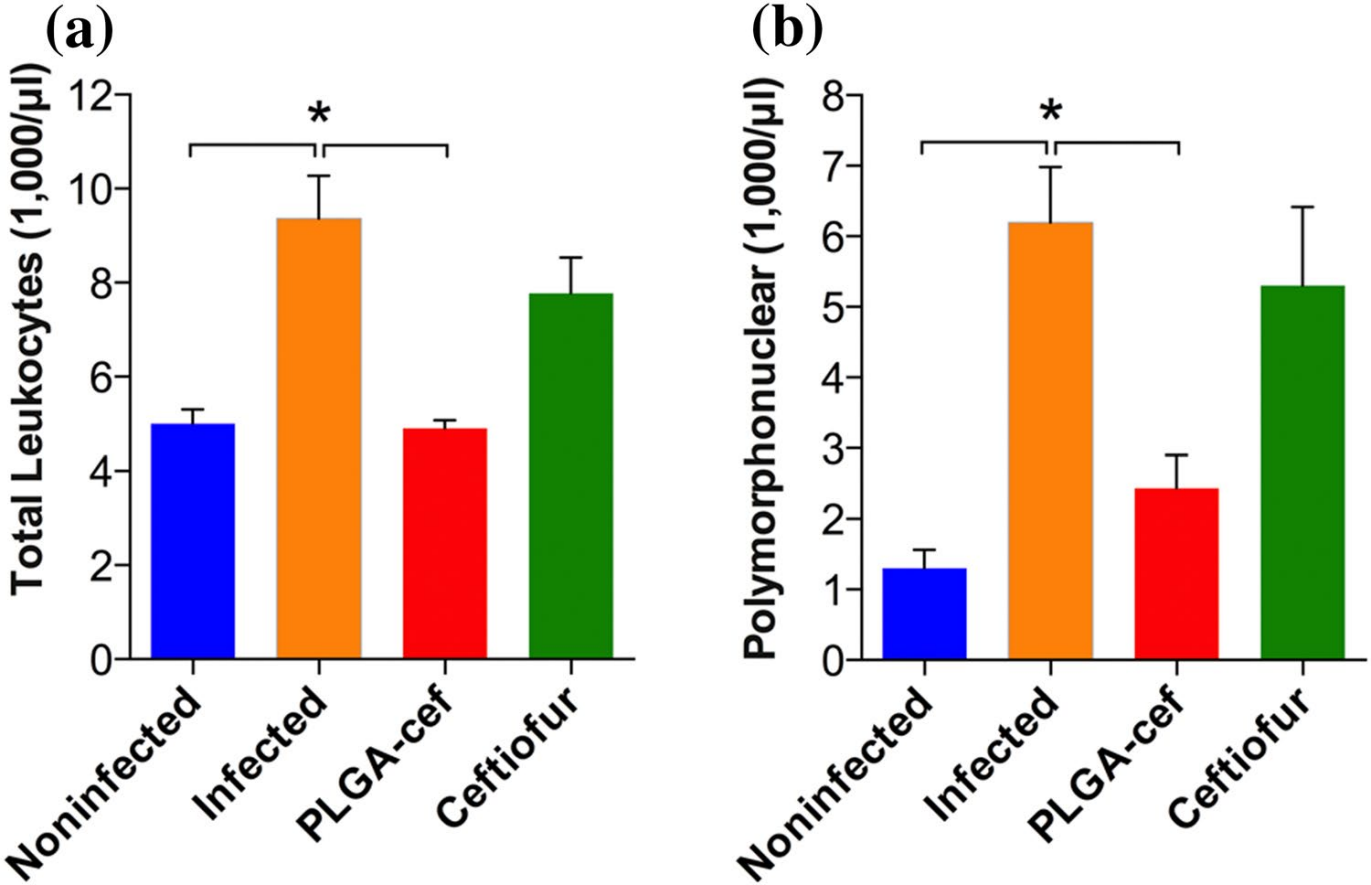
Analysis of hematological blood parameters **a** leukocytes and **b** polymorphonuclears cells, of rats infected with *S. typhimurium*. © 2015 Vilos et al. (2015)

Moreover, the animals treated with cef-PLGA showed hematological parameters similar to the non-infected rats, while the levels of leukocytes and polymorphonuclear cells in the infected animals and in the animals treated with free ceftiofur were higher than those of the non-infected group in a significant way, suggesting that cef-PLGA caused a greater therapeutic effect against *S. typhimurium* (Vilos et al. 2015) (Fig. 4).

As already reported, another innovative application of the MPs involves the use of PLGA MPs incorporated into rapid dissolving microneedle patches (MNPs) for intradermal vaccinations (Angkawinitwong et al. 2020).

The use of MNPs in vaccination is a novel approach that may avoid the obstacles of hypodermic needles and is certainly more tolerated by the patients (Jamaledin et al. 2020a, b).

**Fig. 5:**
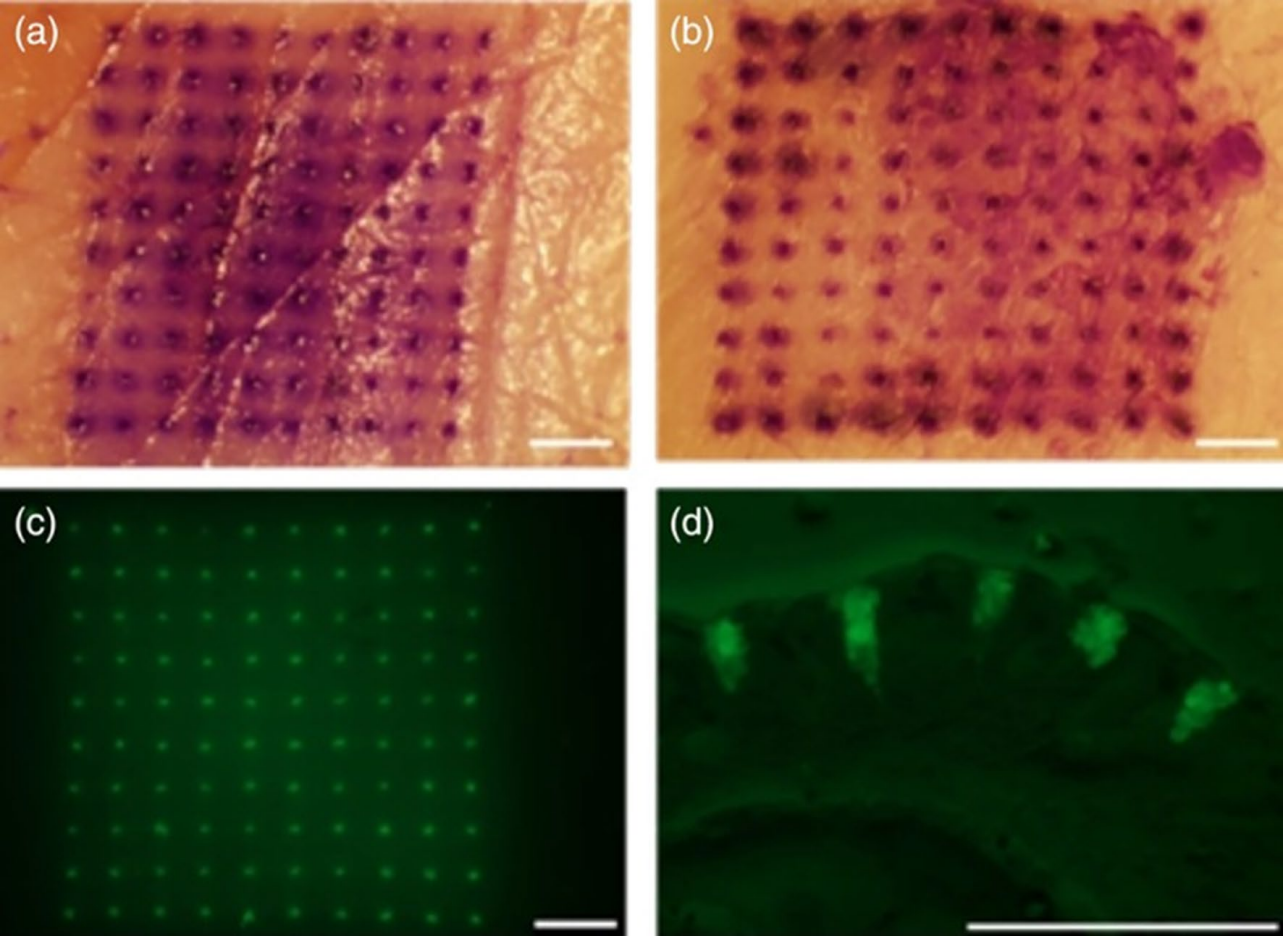
Upper: penetration of microneedle patches in the porcine skin and deposit microparticles intradermally **a** standard patch, **b** pedestal patch; lower: fluorescent micrographs showing the overhead (**c**) and cross-sectional (**d**) view of the skin after application of patch containing fOVA-loaded PLGA MPs. © 2018 Mazzara et al. (2019)

Mazzara et al. (2019) recently developed controlled release PLGA MPs that stably encapsulate different vaccine antigens through aqueous active self-healing encapsulation (ASE). This innovative approach allowed to produce, in a first step, MPs with a protein-trapping agent inside and subsequently a self-healing process that loads the protein inside the MP, thus allowing stable protein to be encapsulated. Interestingly, this technique allows loading multiple vaccine antigens into the same MP. Once formed, these MPs were incorporated in an MNP able to penetrate skin and release MPs that remain in the tissue for a long time (Fig. 5). Interestingly, the measurements of trans‐epithelial water loss (TEWL) over time revealed that murine skin was able to close the micropores after application of the MPs in 96 h.

In vivo experiments, in C57Bl/6 or BALB/c mice confirmed that these patches produce strong immune responses comparable to conventional administration techniques (Mazzara et al. 2019).

The results listed in this section give hope in the real use of these MPs in pharmaceutical field.

## Conclusion

PLGA MPs represent a very simple drug delivery system for different administration routes such as the pulmonary, oral and transderm alone, the latter by the support of MNP that offer a valid tool to penetrate the stratum corneum. PLGA, as described, is the ideal polymer for the preparation of micro- and nanoparticles because of its useful features. In particular, degradation time and morphological characteristics of PLGA MPs can be easily modified by monomer composition, MW and end-group functionalization. By simply modifying PLGA features, it is possible to obtain particles with the desired characteristics and physiological behaviour. One of the most interesting aspects of PLGA MPs is the possibility of encapsulating different kinds of drugs and of preparing a multidrug system using multiple emulsion-based techniques. Moreover, the capability of embedding different kinds of molecules have made PLGA MPs highly valuable for several industrial fields including pharmaceutical, food and cosmetics industry. Amazing advances in the preparation of PLGA MPs have been made in both the emulsion evaporation methods and microfluidics systems, in terms of control of the desired features such as size, shape, and porosity. Both procedures show to be easily compatible with a scale-up production; this aspect, joined to the low cost of the material, makes PLGA MPs truly interesting for pharmaceutic, cosmetics and food industries. However, the best way to obtain a low polydispersity index and a high encapsulation efficacy is to employ membrane emulsification method, and even more microfluidics systems, due to their fine control of the working conditions(i.e. shear stress effect, osmotic pressure, membrane composition) (Field et al. 2017). Among the improvement made in PLGA MP preparation, there is the choice of using no-toxic organic solvent to reduce the risk of toxic residues in the final products and to improve workers security. PLGA MP characteristics should not be underestimated because they affect the drug encapsulation and release; so it is important to select the best conditions to guarantee the desired features. Furthermore, as reported in literature, PLGA MP behaviour could be easily predicted using mathematical drug release models reducing the number of experiments. Finally, preliminary in vivo evaluations gave indication that PLGA MPs could represent the ideal candidates for the pharmaceutical sector.

## Abbreviations

PLGA: Poly(lactic-*co*-glycolic acid)
MPs: Polymeric microparticles
NPs: Polymeric nanoparticles
MW: Molecular weight
PGA: Poly(glycolic acid)
PLA: Poly(lactic acid)
FDA: Food and Drug Administration
EMA: European Medicine Agency
SPG: Shirasu porous glass
PRINT: Particle replication in non-wetting templates
EHDA: Electrohydrodynamic atomization
PEG: Polyethylene glycol
PVA: Polyvinyl alcohol
RM: Rasagiline mesylate
PDI: Polydispersity index
DMSO: Dimethyl sulfoxide
PVP: Polyvinylpyrrolidone
SDS: Sodium dodecyl sulfate
ICH: International Conference on Harmonization
PDE: Permitted daily exposure
W/O: Water in oil
W/O/W: Water in oil in water
LbL: Layer-by-layer
PDMS: Poly(dimethyl siloxane)
PMMA: Poly(methyl methacrylate)
PC: Polycarbonate
SEM: Scanning electron microscope
FESEM: Field emission scanning electron microscopy
CF: Confocal microscopy
OM: Optical microscope
TGA: Thermogravimetric analysis
Tg: Glass transition
DSC: Differential scanning calorimetry
ATR–FTIR: Attenuated total reflectance–Fourier transform infrared
XRD: X-ray diffraction
HBs Ag: Hepatitis B surface antigen
VEGF: Vascular endothelial growth factor
BSA: Bovine serum albumin
DOTAP: 1,2-Dioleoyl-3-trimethylammonium-propane
MNPs: Microneedle patches
PD: Parkinson disease
ROS: Reactive oxygen species
LCAT: Lecithin–cholesterol acyltransferase
ChABC: Nanoparticles loading chondroitinase ABC
CSPGs: Chondroitin sulfate proteoglycans
SCI: Contrast the spinal cord injury
EPR: Enhanced permeability and retention
PTX: Paclitaxel
NRP: Neuropilin
DTX: Docetaxel
HPLGA: HA-coated PLGA
DPPs: Discoidal polymeric particles
VD3: Vitamin D3
HDT: Host-direct therapy
FG_BPC-PLGA-MPs: Bupivacaine-loaded PLGA MPs and fibrin glue
ATRA: All trans-retinoic
TB: Tuberculosis
Mtb: *Mycobacterium tuberculosis*
TEWL: Acid trans‐epithelial water loss
PWT: Paw withdrawal threshold
cef-PLGA: Ceftiofur loaded PLGA MPs
ASE: Active self-healing encapsulation
